# Influence of mating strategies on seminal material investment in crabs

**DOI:** 10.1038/s41598-022-21116-4

**Published:** 2022-11-01

**Authors:** Katrin Pretterebner, Luis Miguel Pardo, Kurt Paschke, Marcela Paz Riveros

**Affiliations:** 1 Programa de Doctorado en Biología Marina, Facultad de Ciencias, Universidad Austral de Chile, 5090000 Valdivia, Chile; 2 Laboratorio Costero de Calfuco, Facultad de Ciencias, Instituto de Ciencias Marinas y Limnológicas, Universidad Austral de Chile, 5090000 Valdivia, Chile; 3 Centro de Investigación de Dinámica de Ecosistemas Marinos de Altas Latitudes (IDEAL), 5090000 Valdivia, Chile; 4 Instituto de Acuicultura, Universidad Austral de Chile, 5480000 Puerto Montt, Chile; 5 Programa de Magister en Didáctica de Las Ciencias Experimentales, Facultad de Ciencias, Pontificia Universidad Católica de Valparaíso, 2340000 Valparaíso, Chile

**Keywords:** Zoology, Animal behaviour, Marine biology

## Abstract

Reproduction involves high energetic costs which are related to behaviour and gamete production. In females energy allocation to gamete production has been well documented. However, estimations of male investment in seminal material are scarce. The present study aims to assess and compare male investment in four brachyuran species by determining biochemical substrates present in the vasa deferentia to subsequently estimate energetic investment during the reproductive cycle. We identified two groups with contrasting energy investments. Two species, *Homalaspis plana* and *Romaleon setosum*, showed high investment due to significant quantities of proteins and lipids. Both species are characterised by large and complex vasa deferentia, and the formation of a remarkably large sperm plug deposited to the female after copulation as a sperm competition avoidance strategy. In contrast, *Metacarcinus edwardsii* and *Taliepus dentatus* invested little energy in their smaller-sized and simpler vasa deferentia. Morpho-functional traits may play a key role in determining the investment, which may also be influenced by mechanisms (i.e. mating tactics) to prevent sperm competition and the intensity of polygyny. This study emphasises the high amount of energy males invest in seminal material and highlights the diversity of mating strategies in Brachyura, which are reflected even on the physiological level.

## Introduction

Investment in reproduction has been recognized to be unbalanced between the sexes due to anisogamy, however males in many animal groups have shown high investment in seminal production at a non-trivial cost^1^. In invertebrates, reproductive activities generally involve considerable energetic costs associated with physiological aspects of gamete production and behaviour^2–4^. For example, in crustaceans, reproductive behaviour is energetically costly, such as pre-copulatory mate guarding in males^5^ or active brooding in females (i.e. ventilation through abdominal flapping)^6^. Revised literature concludes that male costs of sperm production have been underestimated, as they have been assumed to be of small size and limitless amount^7,8^. Seminal material consists of sperm and seminal fluids, which might involve high energetic costs of production^1^ and a slow recovery (e.g.^9^). The composition of ejaculates is generally complex in invertebrates and non-sperm components can assume important functions^10,11^. In some insects, seminal proteins are associated with various functions such as long-term sperm storage in the female receptacle, mating plug formation and ejaculate effectiveness^11–13^. Fatty acids can also be involved in the sperm plug, for example, in the bumblebee *Bombus terrestris*^14^.

In decapods, the male reproductive system consists of paired testes, vasa deferentia and ejaculatory conducts^15^. In the testes, the development of sperm takes place entirely or partially, while in the vasa deferentia sperm are packed into spermatophores, embedded in seminal fluid and stored before mating^15,16^. Consequently, seminal material is stored in the vasa deferentia before delivery to the female during copulation. Thus, the content of vasa deferentia represents the male´s potential reproduction and direct measures of energetic investment should consider its composition. In decapods, seminal fluids delivered by males during copulation to the female can have multiple roles such as sperm nourishment, facilitation of sperm transportation, microbial control or the formation of a sperm plug^17–22^. In brachyuran species, spermatophores and seminal fluids seem to consist principally of proteins and smaller proportions of carbohydrates and lipids^23–25^. Although seminal fluids play numerous roles, the quantitative determination of the biochemical composition of the vas deferens is limited to very few species^23,24,26^.

Brachyuran crabs have highly variable mating strategies which are associated with different levels of polygyny. Intensity of sperm competition, which takes place inside of the seminal receptacle of females, may be a strong driver for reproductive effort in males^27^. For example, species with a high level of polyandry have a heavier reproductive system^28^ which has been detected in fishes, sharks and crickets^29,30^ and, in contrast, monogamous spiders have developed permanent sperm depletion after adulthood^31^. In the case of crabs, in species with fertilization given for last-male precedence^32^, male-male competition is increased, as well as male strategies to assure paternity. Male competitive mating tactics (i.e. morphological, physiological, and behavioural adaptations to avoid sperm competition), such as mate guarding, multiple copulations and sperm plugs, frequently appear when sperm competition is intense^33^. It has also been demonstrated that the intensity of sperm competition depends on the period of receptivity of the female, morphological traits of the female seminal receptacle and male-male agonistic competition abilities^34–36^. The coevolution of the female-male reproductive system in crabs^22^ suggests that the composition and quantity of biochemical components transferred during copulation may be closely linked to the morphology and complexity of the female sperm storage organ and the major functional roles they fulfil after mating in the female. In turn, male reproductive systems should also be complementary to female necessities of amount and features of seminal material.

Therefore, we expected that species that undergo a high level of polygyny and intense sperm competition (i.e. short period of female receptivity) would exhibit a large vas deferens and a large investment of energy in seminal material as these adaptations may help males compete when sperm competition occurs. In temperate regions there is an expected variation in energy investment in reproductive structures of marine invertebrates in order to match the offspring release with cycles of environmental factors like temperature and productivity, therefore seasonal variation in reproduction is very common^37^. Brachyura are ideal model organisms to assess male energetic investment in seminal material, because they exhibit a broad spectrum of reproductive traits and mating strategies which necessarily have an association with the energy involved to develop these strategies. The comparison of various crab species may allow determining patterns among species related to energetic investment in males.

Four brachyuran species, the purple crab *Homalaspis plana*, the hairy crab *Romaleon setosum* (synonymous *Cancer setosus*, *C. polyodon* and *R. polyodon*), the marble crab *Metacarcinus edwardsii* and the kelp crab *Taliepus dentatus*, are among the most important artisanal fishery resources along the Chilean coast. Studies concerning males are almost absent and principally descriptive^26,38,39^ although they are the main target of fishery. The crab species *R. setosum* and *M. edwardsii* belong to the same family (Cancridae). Our model species from other families, Epialtidae (*T. dentatus*) and Platyxanthidae (*H. plana*), are phylogenetically distant from the Cancridae, as shown in the phylogenetic tree for brachyuran families^40^. These four species were chosen as model species because they exhibit contrasting mating strategies, representing a gradient between a continuous mating period and a brief mating season as well as different levels of pre- and/or post-copulatory mate guarding, or the formation of a sperm plug during copulation (comparison of information related to reproduction see Table 1). Also, synchronization of cycles in males and females^41^ is not obvious because Brachyura have a seminal receptacle where after mating females can storage sperm for a long period, but this could depend on the mating strategy (continuous or seasonal mating). The present study aims to assess seasonal patterns of main energetic substrates and energy investment per vasa deferentia during the reproductive cycle in four brachyuran species and associate them to their mating strategies.

**Table 1:** Summary of mating tactics and size and complexity of male and female reproductive systems of the four brachyuran species studied.

Species (family)	Mating period	Mate guarding	Sperm plug	Sperm storage pattern	Vas deferens	Seminal receptacle	References
*Homalaspis plana* (Platyxanthidae)	Autumn	Not present	Present	Large sperm plug, without stratification	Large, complex	Large, very extensible	^26^, LMP (pers. observation)
*Romaleon setosum* (Cancridae)	Spring and summer	Present	Present	*	Large, complex	Large	^39,60^, LMP (pers. observation)
*Metacarcinus edwardsii* (Cancridae)	Spring and summer	Present, prolonged	Present	Clear stratification (sperm gel)	Small, simple	Small, simple	^44,50,57^
*Taliepus dentatus* (Epialtidae)	Year-round	Present, brief	Absent	*	Small, simple	Medium, complex	^51,56,58^

*Refers to not investigated yet.

## Results

### Weight, biochemical components, and energy content of vasa deferentia

**Figure 1:**
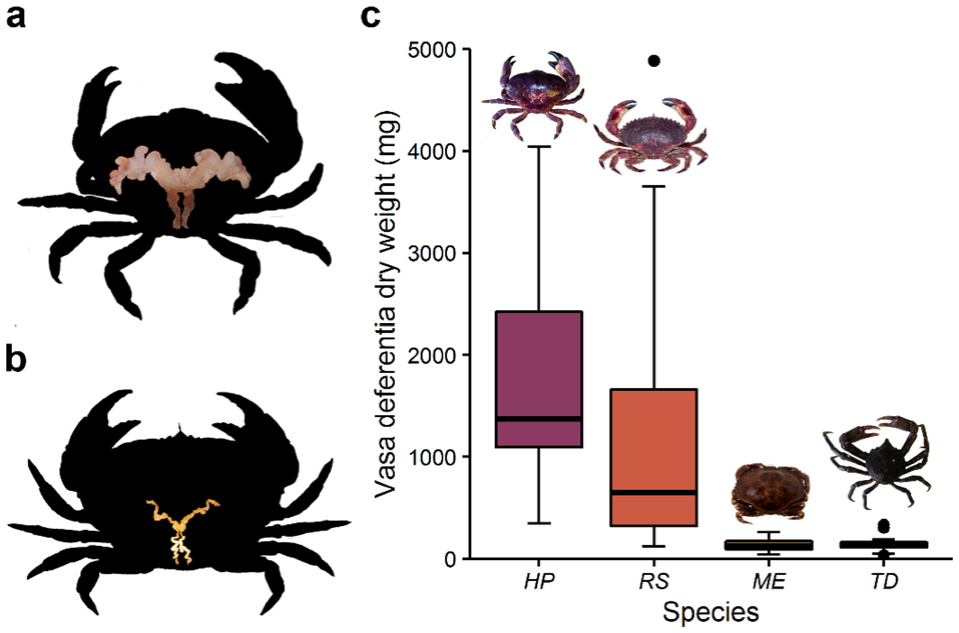
Differences in size and complexity of the vasa deferentia of the brachyuran species studied. (**a**) Large and complex in *Homalaspis plana*. (**b**) Small and simple in *Metacarcinus edwardsii*. (**c**) Box plot of annual dry weight of paired vasa deferentia. *HP*, *RS*, *ME,* and *TD* refer to *H. plana*, *Romaleon setosum*, *M. edwardsii* and *Taliepus dentatus*, respectively. Boxes: interquartile range of the data (first quartile, median and third quartile). Whiskers: the values that extend to 1.5 times the interquartile range. Circles: outliers.

The vasa deferentia of *H. plana* (Fig. 1a and Table 1) and *R. setosum* (Table 1) were extended and highly convoluted (i.e. complex). The vasa deferentia of *M. edwardsii* (Fig. 1b and Table 1) and in particular of *T. dentatus* (Table 1) were of simpler morphology. Annual median vasa deferentia of *H. plana* and *R. setosum* were larger, in comparison to those of *M. edwardsii* and *T. dentatus*, which were relatively small (Fig. 1c).

**Figure 2:**
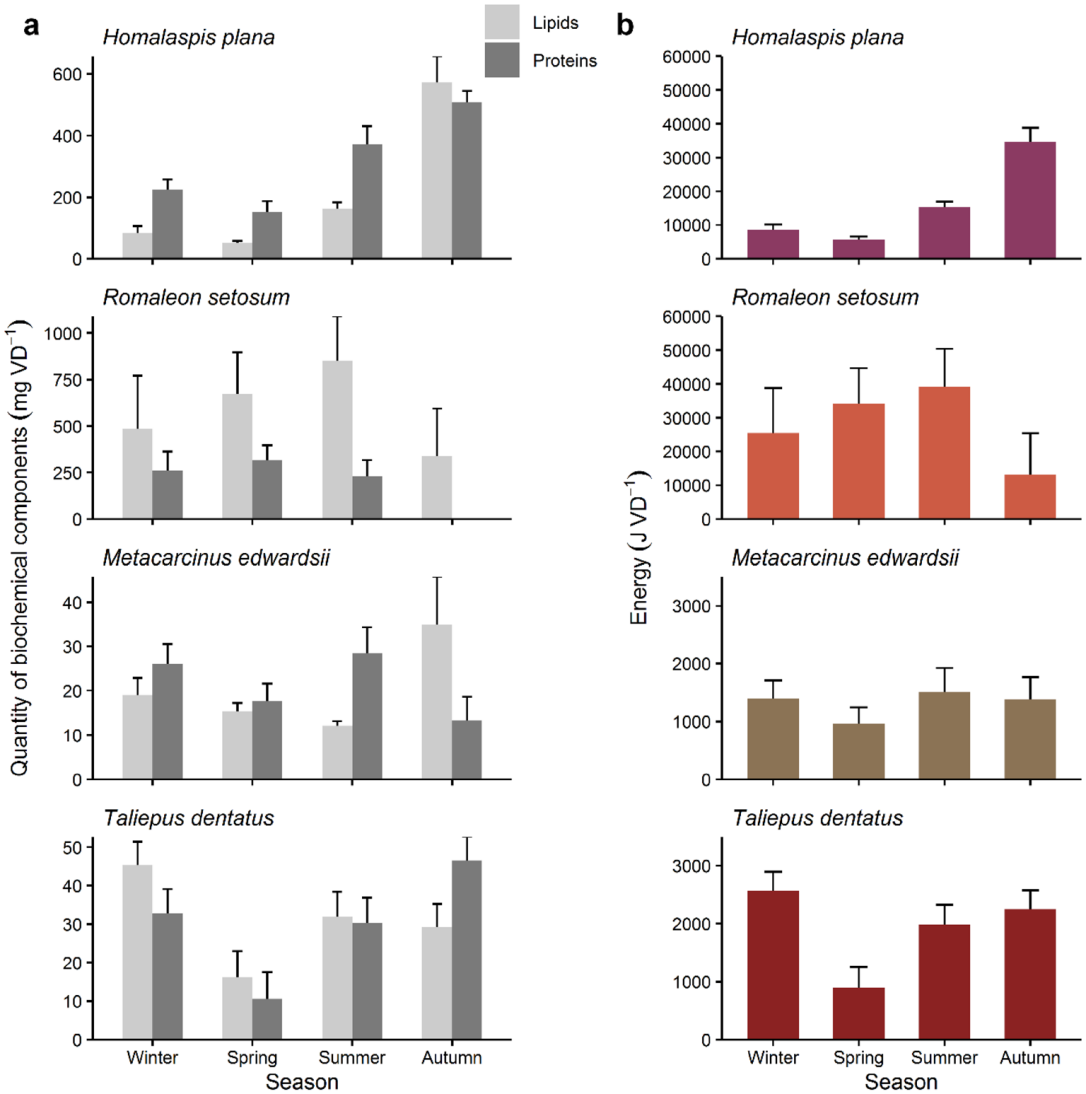
Seasonal pattern of biochemical components and energy present in the paired vasa deferentia of the four brachyuran species studied. (**a**) Quantity of proteins and lipids. Note different scale for each species. Values correspond to adjusted means + *SE* in all species, except for quantity of proteins and lipids in *Homalaspis plana* and lipids quantity in *Metacarcinus edwardsii* which correspond to means + *SE*. Seasons correspond to austral seasons. (**b**) Energy content. Note different scales of energy investment of *H. plana* and *R. setosum*, and *M. edwardsii* and *Taliepus dentatus*. Values correspond to adjusted means + *SE* in all species, except for in *H. plana* which correspond to means + *SE*. Energy content was estimated by converting amounts of lipids and proteins to their caloric equivalents. Seasons correspond to austral seasons.

In *H. plana* significant seasonal changes in the quantity of proteins and lipids revealed a similar pattern: decreased values during winter and spring, followed by a continuous increase in summer and autumn (Table 2 and Fig. 2a). Quantity of proteins was generally at least twice the amount of lipids in each season; apart from in autumn quantity of both components was similar. In *R. setosum* no significant fluctuations in the quantity of proteins and lipids were detected (Table 2). However, a trend of increased lipids quantity from winter until summer was observed, and in autumn, the quantity of lipids seemed to decrease (Fig. 2a). The amount of proteins remained relatively constant from winter until summer and dropped to zero in autumn. In *M. edwardsii* only the quantity of lipids fluctuated significantly seasonally (Table 2). The quantity of lipids decreased successively from winter until summer and increased in autumn (Fig. 2a). The quantity of proteins was slightly diminished during spring, increased during summer, and was declined again during autumn (Fig. 2a). In *T. dentatus* quantities of proteins and lipids varied significantly seasonally (Table 2). Quantities of both proteins and lipids decreased in spring and increased during summer (Fig. 2a). Compared with summer, quantity of lipids remained constant in autumn, while proteins increased.

**Table 2:** Permutation tests of the linear model comparing the effects of size (covariate) and season (fixed factor) on quantity of each biochemical component and energy in crab species.

Factor	*df*	MS	No. of iterations	*P*
**Homalaspis plana**				
Quantity proteins				
Season	3	1.23304 × 10^5^	5000	** < 0.001**
Residuals	15	9.056 × 10^3^		
Quantity lipids				
Season	3	2.83779 × 10^5^	5000	** < 0.001**
Residuals	15	1.0884 × 10^4^		
Energy				
Season	3	8.30444528 × 10^8^	5000	** < 0.001**
Residuals	15	3.0645819 × 10^7^		
**Romaleon setosum**				
Quantity proteins				
Size	1	4.99166 × 10^5^	5000	** < 0.001**
Season	3	7.5455 × 10^4^	1259	0.105
Residuals	13	3.1481 × 10^4^		
Quantity lipids				
Size	1	2.108463 × 10^6^	5000	**0.006**
Season	3	2.18988 × 10^5^	233	0.613
Residuals	13	2.37098 × 10^5^		
Energy				
Size	1	5.493224945 × 10^9^	5000	**0.007**
Season	3	5.68766463 × 10^8^	292	0.387
Residuals	13	5.32254899 × 10^8^		
**Metacarcinus edwardsii**				
Quantity proteins				
Size	1	6.71 × 102	5000	**0.004**
Season	3	1.0654 × 102	473	0.289
Residuals	14	7.89 × 101		
Quantity lipids				
Season	3	512.98 × 102	2792	**0.034**
Residuals	15	173.92 × 102		
Energy				
Size	1	1.874968 × 106	3422	**0.028**
Season	3	2.66510 × 105	360	0.611
Residuals	14	4.00560 × 105		
**Taliepus dentatus**				
Quantity proteins				
Size	1	1.85274 × 103	5000	**0.006**
Season	3	9.9857 × 102	5000	**0.008**
Residuals	15	1.9207 × 102		
Quantity lipids				
Size	1	1.71827 × 103	5000	**0.01**
Season	3	5.9521 × 102	5000	**0.04**
Residuals	15	1.7943 × 102		
Energy				
Size	1	7.057318 × 106	5000	**0.001**
Season	3	2.215233 × 106	5000	**0.007**
Residuals	15	5.15481 × 105		

Bold indicates significant differences (*P* < 0.05).

Note that analyses of *Homalaspis plana* and quantity of lipids of *Metacarcinus edwardsii* do not include size as a covariate.

In *H. plana,* the energy content showed significant seasonal variation (Table 2). Energy content in *H. plana* was very low in winter and spring and increased until reaching a maximum mean value of 34,618 J in autumn (Fig. 2b). In *R. setosum* no significant seasonal energy fluctuations were detected (Table 2). Energy content increased successively from 25,415 J in winter until reaching the highest value of 39,177 J in summer (Fig. 2b). Compared with summer, energy decreased approximately two-thirds in autumn to a minimum mean value of 13,138 J. In *M. edwardsii* no seasonal fluctuations in energy content were detected (Table 2), whereby spring energy values were slightly lower (Fig. 2b). In *T. dentatus,* energy invested per vasa deferentia varied significantly seasonally (Table 2). During spring, energy decreased more than half compared with each of the other seasons (Fig. 2b).

### Comparison among species

**Figure 3:**
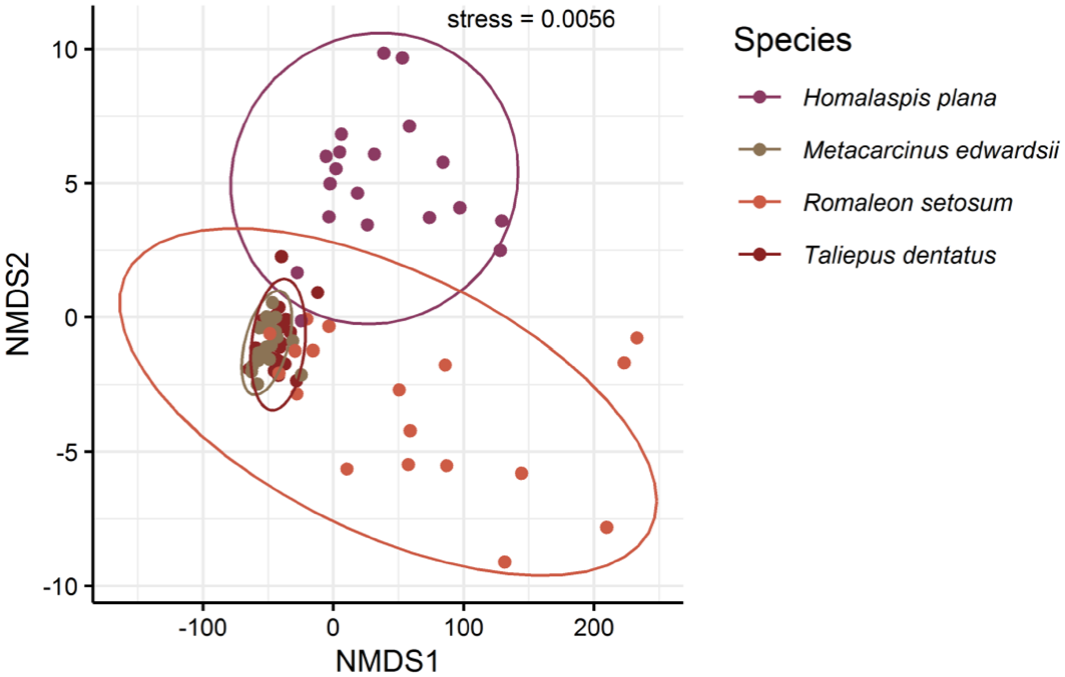
Non-metric multidimensional scaling (nMDS) plot consisting of seasonal data of four brachyuran species. Non-metric multidimensional scaling is a tool to assess similarity between samples when considering multiple variables of interest. The value stress represents how well points fit within the specified number of dimensions (stress < 0.05 indicates good fit). The variables included in the nMDS were quantities of proteins and lipids, the vasosomatic index and the total energy content per paired vasa deferentia. Data were square root transformed for analysis.

In the nMDS *H. plana* was displayed as a group relatively separated from the other species (Fig. 3). The relatively scattered cluster of points corresponded to *R. setosum* and was probably attributed to the great seasonal fluctuation of values. The two species, *M. edwardsii* and *T. dentatus*, were depicted aggregated together probably due to smaller seasonal variations.

## Discussion

Two groups with large differences in complexity of the vas deferens and energy investment were clearly distinguished in the studied crabs, suggesting divergent strategies in the evolution and development of the male reproductive system. The crab species *H. plana* and *R. setosum* showed high investment in terms of energy in the male seminal storage structure, which was larger and more complex, in contrast to the reproductive system of *M. edward*sii and *T. dentatus*. Interestingly, two species (*R. setosum* vs. *M. edwardsii*) from the same family (i.e. Cancridae) showed a contrasting evolution in the size of the male reproductive system, evidencing no phylogenetic restriction in this matter. The greatest contrast in the morphology of the vas deferens was between species from the same family (Cancridae) which suggests that the effects of the evolutive history did not influence the results. Moreover, energetic investment in male reproduction seems to be well associated with the specific mating strategy composed of several mating tactics (Table 1).

Several tactics have been developed in male crabs to avoid sperm competition, such as sperm plugs and stratification of ejaculates, both with great use of non-sperm material. Sperm plugs serve as a paternity assurance device that prevents subsequent inseminations by other males^17,42,43^. This appears as a highly effective strategy that can lead to genetic monogamy even in the presence of polyandry^44^. The seminal receptacle of post-copulated females in *H. plana* and *R. setosum* is characterized by the presence of a large sperm plug^26,39^ and a significant part of the biochemical components of seminal material delivered by males may be involved in its formation. The biochemical composition of the sperm plug itself is known in a few decapod species^45^. For example, in the portunid *Arenaeus cribrarius,* the sperm plug inside of the seminal receptacle of mated females consists of glycoprotein^46^. Another example is the sperm plug of *C. danae* which is composed of a layered strata of three types of secretions, each of a chemically distinct composition of proteins and polysaccharides, which are produced by males through the combined secretions from the three vas deferens regions^47^. In the sperm plug of distinct insect species, proteins and lipids have been detected^14,48,49^. For example, in *Drosophila* protein contributes to forming of the plug^49^, but in the bumblebee *B. terrestris* the active substances are fatty acids^14,48^. The presence of a sperm plug may increase the probability of paternity^32^, and males may benefit by increasing investment in a single female. Though, investment in a sperm plug and mating with only one female could lead to ejaculate depletion. Recently, it has been suggested that males of *H. plana* are likely able to mate only once during their reproductive period due to the great amount of ejaculate received by the female seminal receptacle^26^. In *M. edwardsii* seminal fluids transferred by the male form a sperm plug which is very discrete and only blocks the narrow vaginal lumen to avoid further matings with rival males^50^. In contrast, males of *T. dentatus* do not produce a plug^51^.

Seminal fluids may play an important role in sperm stratification, which is another mechanism with which males can influence fertilization in their favour through clearly separating ejaculates transferred by different mates and reducing mixing of sperm^52,53^. The exact biochemical composition of sperm gel in crabs has been poorly studied. For example, in the seminal receptacle of the majoid *Stenorhynchus seticornis* stratified sperm packets are surrounded by secretions that consist mainly of proteins, combined with acid and neutral polysaccharides^54^. In *M. edwardsii,* proteins and lipids in the vas deferens may play an important role in avoiding sperm competition in the form of sperm gel, which separates sperm received by different males, thereby allowing a clear stratification of sperm in the receptacle^50^. In the ventral-type seminal receptacle of *M. edwardsii* new ejaculates displace old ones toward the blind end of the receptacle and are deposited closer to the oviduct connection promoting last-male precedence^32^. In *R. setosum* the storage pattern of ejaculates in the seminal receptacle has not been investigated yet, but in the family Cancridae in which females may mate with various males, sperm stratification has been reported in several species and seems to be widespread^50,55^. In *T. dentatus* the biochemical substrates transferred may contribute to sperm gel in the seminal receptacle for embedding and storage of free sperm^56^. In *T. dentatus* it has not been investigated yet, whether sperm masses are mixed or stratified in the dorsal chamber of the two-chambered receptacle. In the analysed seminal receptacle of *H. plana* a continuum of sperm gel and spermatophores has been observed in the blind end of the sac, which suggests no stratification of ejaculates (LMP, pers. observation).

The quantity of each biochemical component and energy stored in the vas deferens may be influenced by the amount of ejaculate transferred in one mating event and particularly the mating frequency. Males of *H. plana* likely mate only once during the reproductive season^26^, thus inverting their entire reproductive energy in only one female. In *M. edwardsii,* lipids in the vasa deferentia decreased during the reproductive season; however, reductions were of small amount probably because males deliver on average only 15% of their vasa deferentia weight in one mating event^57^ and portion their seminal material to various females (high level of polygyny^44^). In *T. dentatus*, males transfer on average 37% of their initial seminal material stock during one mating^51^. The imminent high risk of sperm competition and uncertainty of paternity may explain the strategy of allocating relatively little energy and small percentage of the initial seminal material stock in each female compared to *H. plana* and increasing chances of paternity through partitioning seminal material among various females (high level of polygyny: 42% of males mate multiply under a female-biased sex ratio^58^). Therefore, in *T. dentatus* the decrease of proteins and lipids probably corresponds to multiple matings indicating increased reproductive activities especially in spring. This suggests that a high level of polygyny (*M. edwardsii* and *T. dentatus*) may be associated with investing little energy in each female but engaging in frequent copulations. The frequency of mating of males of *R. setosum* has not been investigated yet.

In addition to the anatomical adaptation to avoid sperm competition, mate guarding is an effective behavioural adaptation to ensure paternity. Post-copulatory guarding has often been observed in species in which receptivity of the female is restricted to a short time after its moult (i.e. female ‘soft-shelled’ during mating)^44,59^. During this vulnerable state, females may also benefit from protection by males from predators, cannibalism or other males^59^. Mate guarding may also be associated with the necessity of protecting the valuable energy invested. Guarding is particularly common in the family of the cancrids^44,55,59^, such as in *R. setosum* and *M. edwardsii*. Males of *R. setosum* perform copulatory guarding approximately one week before the females’ moult and 2–3 days post-copula^60^. Especially for males of *R. setosum,* which spent large quantities of energy on reproduction, it may be crucial to protect their investment through guarding. In *M. edwardsii* pre- and post-copulatory mate guarding is extended^44^. In the presence of rival males the duration of guarding in males of *M. edwardsii* is increased; thus, it serves as a strategy against sperm competition and provides better protection for their energy invested. In the case of *H. plana*, only precopulatory guarding has been detected and probably the large sperm plug present in this species is enough to ensure paternity. In *T. dentatus* both pre- and post-copulatory mate guarding is brief^51^. Likely in *T. dentatus,* relatively low energetic investment in reproduction does not require extended protection of the ejaculate after mating as well as females are receptive year-round which may relax male-male competition. Mating with ‘hard-shelled’ females, energy-conserving short mate-guarding and the absence of costly prevention of competition (e.g. no sperm plug) may facilitate males of *T. dentatus* to recover seminal reserves fast (within 15 days)^61^ and to increase the mating frequency. Although males of *H. plana* inverted the greatest quantity of energy in reproduction of the four species studied, this species normally does not perform post-copulatory guarding (LMP, pers. observation). Probably the large internal sperm plug is sufficient to efficiently protect the ejaculate, prevent further pairings and ensure paternity.

*Homalaspis plana* and *R. setosum* invested remarkably high energy in their vasa deferentia, reaching maxima of nearly 40,000 J (autumn and summer, respectively). The energy peak in each of the two species was followed by a sharp decline in both. The pattern of seasonal energy fluctuations may be indicative of the reproductive period of a species. An abrupt decrease of energy may coincide with the mating period or the end of it. In *H. plana* the decrease of ~ 80% of energy in winter compared with autumn probably indicates that matings have occurred shortly before this period. Energy remained low in spring and successively increased during summer and autumn. In *R. setosum* the abrupt decrease of two-thirds of the energy in autumn compared with summer likely indicates that males transferred seminal material to females just before this period and recovering values from winter until summer. The presence of ovigerous females of *R. setosum* was observed throughout the austral winter until larvae hatching in spring in a similar latitude^60^, whereby in both species, mating occurs when the cycle of the ovary development begins (as has been detected in other crab species^46,50,62^) and a high quantity of proteins in the seminal fluid delivered to the female seminal receptacle could function as a cue for gonadal development. Seminal fluid proteins (SFPs) produced in the male reproductive system are well recognized to induce numerous physiological and behavioural post-mating changes in female insects, such as egg production^13^. However, SFPs have been poorly studied in decapods^63^. Other key functional properties associated with the great quantity of biochemical components in the vas deferens may be related to mechanisms that increase the reproductive return. In *R. setosum* male seminal fluids may be involved in enhancing sperm viability during prolonged storage within the female seminal receptacle as this species is able to produce several viable clutches without re-mating^60^. There is evidence from females of *M. magister,* which store viable sperm for at least 2½ years in their seminal receptacle, which can be used to inseminate more than one egg mass^64^. Especially proteins have been formerly associated with this function in insects^12^; however, lipids may also be valuable in prolonged sperm storage due to their high energetic value. Beyond that, lipids may serve as a contribution of energy to the female in *H. plana* and *R. setosum* due to the great amount of this highly energetic component. In insects, males commonly transfer gifts that contain many different non-gametic materials to females during courtship and mating^65^. In this way, the provision of nutrients deposited in the female’s reproductive tract can occur.

**Figure 4:**
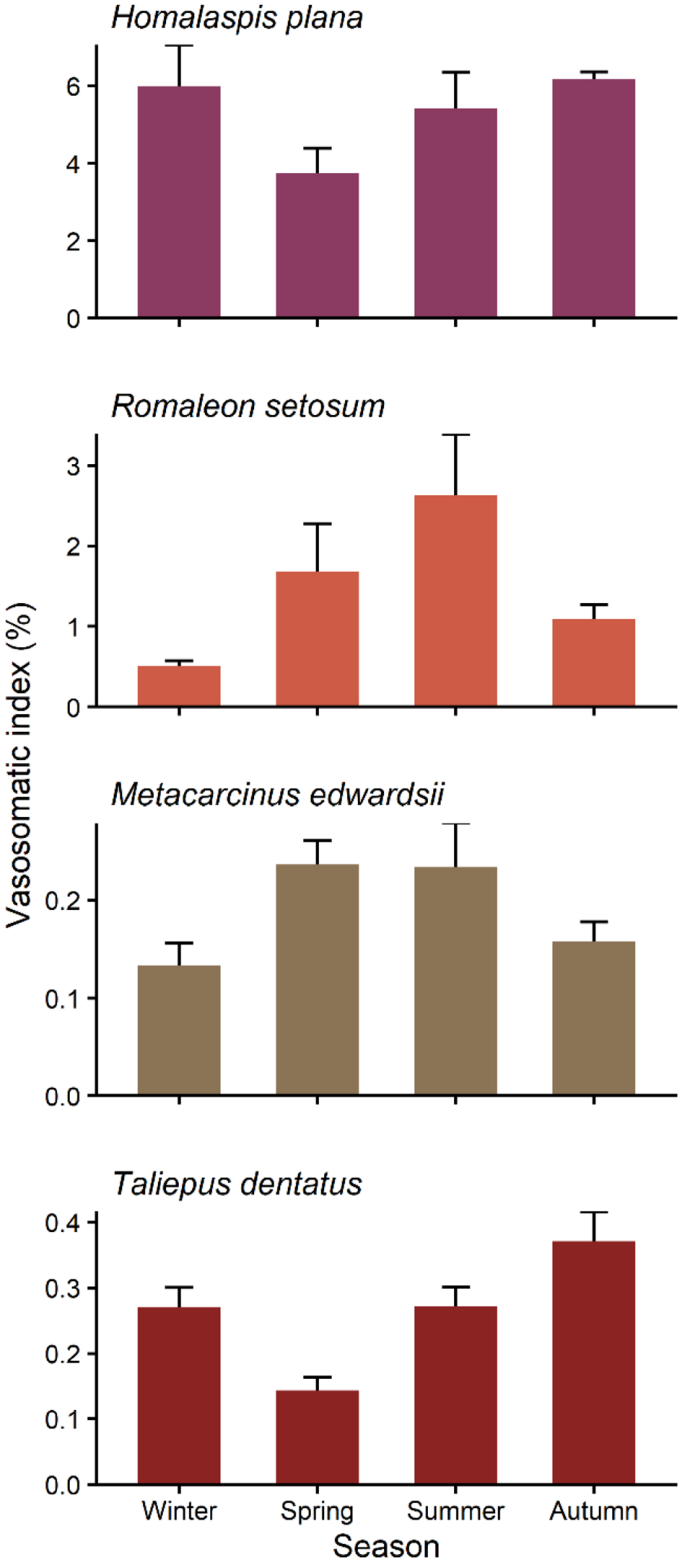
Seasonal pattern of vasosomatic index of the four brachyuran species studied. Values correspond to means + *SE*. Seasons correspond to austral seasons.

While *H. plana* and *R. setosum* assigned similarly high amounts of energy, the energy invested by *H. plana* was linked to the production of large amounts of proteins and lipids, in contrast to *R. setosum,* which produced highly energetic lipid-rich vasa deferentia. In *H. plana* the vasa deferentia dry weight as a proportion of the crab’s whole body dry weight accounts for up to nearly 6% displaying one of the largest VSI recorded so far in crabs^26^ (Fig. 4). This emphasizes the large energy expenditure, which may result from the capacity of the vas deferens to produce and store large quantities of proteins and lipids. In this species, especially the voluminous posterior region of the vas deferens has been identified as the main site of seminal fluid production with a very high energy content^26^. The main energetic substrates in *H. plana* were proteins and lipids, and in winter, quantities of lipids and proteins dropped nearly 90% and two-thirds, respectively, compared with autumn. These seasonal fluctuations indicate that males delivered large quantities of both components during mating to the female. Especially in *H. plana* quantities of proteins and lipids remained relatively low during winter and spring. Recovery may require a long period after mating suggesting that the elaboration of these large amounts of proteins and lipids may have elevated energetic costs. In this context, interesting future research questions may arise, such as whether the elaboration of specific components or the composition of the seminal reserves influences the recovery period after mating.

The crab species *M. edwardsii* and *T. dentatus* showed low energetic investment per vasa deferentia. They had similar patterns of investment with relatively constant energy values throughout the seasons, except for in austral spring the lowest energy values (around 900 J) were recorded in both species. In *M. edwardsii* the reduction of energy coincides with the well-known mating season from October to January (i.e. austral spring and summer)^50^. In *T. dentatus* the marked decline of energy content during spring corresponds to the formerly described continuous reproductive pattern but with a greater mating intensity in spring^51^. Both species with low male energy investment were characterized by smaller-sized vas deferens and intermediate VSIs (Fig. 4). Annual mean vasa deferentia dry weight of *M. edwardsii* and *T. dentatus* only accounted for approximately 10% of those of *H. plana* and *R. setosum*. While the two crab species show a similar investment in terms of energy, this is likely related to the mating strategies.

Complex and well-developed male reproductive systems appear to have evolved at different times in the evolution of crabs as is noted in a contrasting composition and size of the vas deferens within one family and similarities between phylogenetically not closely related species. Thus, the composition and size of the vas deferens in crabs is related to the mating strategy and level of polygyny of each species. To summarize, the morpho-functional traits of the reproductive system and competition avoidance strategies may interact to determine the optimal mating frequency and the quantity of biochemical components, which can also be reflected in the energy invested (Table 3). Energy allocation may also be associated with the intensity of polygyny of a species. We suggest that a gradient of the intensity of polygyny exists related to energetic investment: allocation of great energy amount and low mating frequency, in contrast to low energetic investment and multiple matings. Interestingly, our findings of energy investment in reproduction resemble the two distinct hypotheses concerning the evolution of large testes stated by Vahed and Parker^28^: the numerical sperm competition hypothesis may correspond to *H. plana* and *R. setosum* vs. the male mating rate hypothesis matches our findings of *M. edwardsii* and *T. dentatus*. Especially in the face of sperm limitation (number of sperm is insufficient to fertilize all oöcytes produced by females) as triggered by size-selective male fishery management^66^, it is crucial to evaluate the ejaculate as a whole because males may be depleted of specific biochemical components of the seminal material^11^. Depletion of distinct biochemical components may reduce the functionality of particular components and affect the reproductive success. Thus, ejaculate limitation rather than sperm limitation could affect fished species. Moreover, in the aquaculture industry, biochemical composition and the quantity of each component may be important indicators for the quality of seminal material. Our study is a first step towards identifying investment in the male seminal storage structure in crabs which highlights the diversity of mating strategies in Brachyura which is reflected even on the physiological level.

**Table 3:** Summary of energy investment in the four brachyuran species studied. The complexity of the reproductive system, intensity of mating, polyandry (based on observations in the laboratory) and existence of a strategy to prevent competition (i.e. sperm plug) are indicated for each species and may play key roles in energy investment.

Species	Energy investment	Complexity reproductive system	Polygyny	Polyandry	Sperm plug
*Homalaspis plana*	+	+	−	−	+
*Romaleon setosum*	+	+	*	*	+
*Metacarcinus edwardsii*	−	−	+	+	+
*Taliepus dentatus*	−	−	+	+	−

*Refers to not investigated yet.

## Methods

### Species sampling and experimental procedures

Males of the following four crab species, *H. plana, R. setosum*, *M. edwardsii* and *T. dentatus* were collected seasonally (austral seasons) from Los Molinos Bay, Southern Chile (39° 51′ 16.7′′ S; 73° 23′ 40.3′′ W) from June 2016 to May 2017 and transported to the Laboratorio Costero de Recursos Acuáticos de Calfuco, Universidad Austral de Chile. Crabs were maintained in the laboratory and provided running seawater, air supply and ad libitum food (mussels). All crabs were measured along their greater body axis; carapace width (CW) in *H. plana*, *R. setosum* and *M. edwardsii* and carapace length (CL) in *T. dentatus*. Mean ± *SE* size of *H. plana*, *R. setosum*, *M. edwardsii* and *T. dentatus* were 94.3 ± 1.4 mm CW, 128.6 ± 3.1 mm CW, 133.9 ± 2.9 mm CW, and 102.7 ± 1.6 mm CL, respectively. No difference in size among seasons existed in each species except for *M. edwardsii* (one-way ANOVA, season: *F*_3, 15_ = 10.05, *P* < 0.001; smaller sized crabs in summer). Each crab species and season comprised five replicates (except for *n* = 4 each in *M. edwardsii* in winter, *H. plana* in winter, and *R. setosum* in winter and autumn).

Crabs were anesthetised (thermal shock − 20 °C for 15 min) and paired vasa deferentia were extracted. Samples were immediately frozen and stored in pre-weighed vials at − 80 °C in an ultrafreezer (Thermo Scientific™, Forma Series 700) until further analyses. The left vas deferens was used for biochemical analyses and the right vas deferens was used to determine the dry weight to relate the quantity of each biochemical component to it. Normally both vasa deferentia have similar weight^67^. Dry weight was determined by oven drying the right vas deferens for 4 days at 70 °C and weighing it to a precision of 0.00001 g. Total dry weight of paired vasa deferentia (i.e. doubling dry weight of right vas deferens) is referred to as VDW. The vasosomatic index (VSI, expressed as percentage) was calculated: VSI = (VDW/BDW) × 100, BDW being the dry weight of the body (oven-dried for 4 days at 70 °C and weighed to a precision of 0.01 g). To estimate the VSI, dry weight of crabs without legs and chelae were used to increase accuracy^22,57^.

### Biochemical analyses

Samples were analysed for total protein and lipid content at the Laboratorio de Ecofisiología de Crustáceos, Universidad Austral de Chile, in Puerto Montt, Chile. Stored samples were lyophilised (Labconco, FeeZone 2.5) for 48 h, and dry weight was determined (precision of 0.00001 g). Samples were homogenised in a phosphate buffer and Milli-Q ultrapure water to achieve a concentration of ~ 10 mg mL^−1^ by a motorised homogenizer (Ultra-Turrax); during this process, samples were kept on ice. Complementary ultrasound (Branson, Sonifier, Cell Disruptor B 15) was applied in pulses of 4 s and 4 s rest (cycle repeated four times maximum to avoid warming) on tissues difficult to homogenize. Each homogenate was subdivided into new cryovials (proteins: 10 µL (3 µL per duplicate in Lowry method) and lipids: 50 µL in sulfo-phospho-vanillin method) and stored frozen at − 80 °C until respective analysis.

Protein content was assessed in duplicate by the Lowry method^68^ using a commercial kit (DC Protein Assay Kit, Bio Rad; Standard BSA; 750 nm). Total lipid content was estimated in duplicate by the sulfo-phospho-vanillin method (Standard cholesterol; 530 nm)^69^, modified for microplate format by Torres et al.^70^. A PowerWave HT spectrophotometer (BioTek) was used for all colorimetric methods. The biochemical composition was calculated based on the calibration curves and values of absorbance. Quantities of biochemical components and dry weight of the right vas deferens were doubled to refer to the paired vasa deferentia. Results were expressed as the averaged quantity of each biochemical component per paired vasa deferentia. The energy content in vasa deferentia of crabs was estimated from the protein and lipid data and was converted to their caloric equivalents in Joule corresponding to 23.64 J/mg protein and 39.54 J/mg lipid^71^. In preliminary analyses quantity of carbohydrates was estimated by the phenol–sulphuric method adapted for microplate format following DuBois et al*.*^72^ (Standard Glucose; 490 nm). The quantity of carbohydrates was very small therefore, they were not estimated in subsequent samples.

### Data analyses

To test seasonal variation in quantity of biochemical components and energy invested in the vasa deferentia in each species, one-way ANCOVAs with permutation were performed with season as a fixed factor and male size as a covariate. However, when the covariate was not significant, a one-way ANOVA with permutation was performed instead. Analyses were chosen because they are distribution-free and allow analysis with relatively small sample sizes. The ‘Aovp’ function in the package ‘lmPerm’ was used^73^. To check for size similarities among seasons, a one-way ANOVA was performed for each species.

Non-metric multidimensional scaling (nMDS) was performed to visualize the pattern of energy investment in seminal material and to identify species with similar investment strategies. It is a tool to assess similarity between samples when considering multiple variables. Data comprised seasonal values of each of the four brachyuran species. The variables included in the nMDS were quantities of proteins and lipids, VSI and the total energy content per vasa deferentia. The ‘isoMDS’ function in the package ‘MASS’ was used^74^. All statistical analyses were performed in R v. 3.5.3^75^.

## Data accessibility

The datasets generated during the current study are available in the Zenodo open science repository, https://doi.org/10.5281/zenodo.5654807^76^.
